# Outbreak of Middle East respiratory syndrome coronavirus in Saudi Arabia: a retrospective study

**DOI:** 10.1186/s12879-016-2137-3

**Published:** 2017-01-05

**Authors:** Fadilah Sfouq Aleanizy, Nahla Mohmed, Fulwah Y. Alqahtani, Rania Ali El Hadi Mohamed

**Affiliations:** 1 College of Pharmacy, Department of Pharmaceutics, King Saud University, 22452, Riyadh, 11495 Saudi Arabia; 2 College of Medicine, Princess Nourah Bint Abdulrahman University, Riyadh, 12484 Saudi Arabia; 3 College of Science, Princess Nourah Bint Abdulrahman University, Riyadh, 12484 Saudi Arabia; 4 Scientific research, Federal Ministry of Health, Khartoum, 303 Sudan

**Keywords:** Middle East respiratory syndrome, Epidemiology, Saudi Arabia

## Abstract

**Background:**

The Middle East respiratory syndrome (MERS) is proposed to be a zoonotic disease. Dromedary camels have been implicated due to reports that some confirmed cases were exposed to camels. Risk factors for MERS coronavirus (MERS-CoV) infections in humans are incompletely understood. This study aimed to describe the demographic characteristics, mortality rate, clinical manifestations and comorbidities with confirmed cases of MERS-CoV.

**Methods:**

Retrospective chart review were performed to identify all laboratory-confirmed cases of MERS-CoV in Saudi Arabia who reported to the Ministry of Health (MOH) of Saudi Arabia and WHO between April 23, 2014 and August 31, 2015. Patients’ charts were also reviewed for demographic information, mortality, comorbidities, clinical presentations, health care facility and presented with descriptive and comparative statistics using non parametric binomial test and Chi-square test.

**Results:**

Confirmed cases of male patients (61.1%) exceeded those of female patients (38.9%). Infections among Saudi patients (62.6%) exceeded those among non-Saudi patients (37.4%; *P* = 0.001). The majority of the patients were aged 21–40 years (37.4%) or 41–60 years (35.8%); 43 (22.6%) were aged >61 years, and (8) 4.2% were aged 0–20 years. There was a difference in mortality between confirmed MERS-CoV cases (63.7% alive versus 36.3% dead cases, respectively). Furthermore, fever with cough and shortness of breath (SOB) (*n* = 39; 20.5%), fever with cough (*n* = 29; 15.3%), fever (*n* = 18; 9.5%), and fever with SOB (*n* = 13; 6.8%), were the most common clinical manifestations associated with confirmed MERS-CoV cases.

**Conclusion:**

MERS-CoV is considered an epidemic in Saudi Arabia. The results of the present study showed that the frequency of cases is higher among men than women, in Saudi patients than non-Saudi, and those between 21 to 60 years are most affected. Further studies are required to improve the surveillance associated with MERS-CoV to get definite and clear answers and better understanding of the MERS-CoV outbreak as well the source, and route of infection transmission in Saudi Arabia.

## Background

Coronaviruses are large enveloped viruses with a single-stranded positive-sense RNA genome. They can infect humans, as well as a variety of animals, such as bats, mice, birds, dogs, pigs, and cattle, causing mainly respiratory and enteric diseases [1]. The virus MERS-CoV is a new member of the beta group of coronavirus, Beta coronavirus. MERS-CoV is different from SARS coronavirus and different from the common-cold coronavirus and known as endemic human betacoronaviruses HCoV-OC43 and HCoV-HKU1. MERS-CoV had frequently been referred to as a SARS-like virus, or the novel coronavirus until 23 May 2013. On September 11, 2012, a 49-year-old man from Qatar, with a history of travel to Saudi Arabia, was transferred to the United Kingdom with symptoms of severe respiratory illness. Sample from the lower respiratory tract samples of the patient was found positive after a pan-coronavirus RT-PCR assay. Comparison of the sequence of the PCR fragments with the ones obtained in the case of the Saudi patient revealed that they share 99% similarity, suggesting infection by the same virus [1]. Sequencing of the novel coronavirus was performed at the Erasmus Medical Center (EMC) in Rotterdam, the Netherlands, where the virus was named “human coronavirus EMC” (hCoV-EMC) [2]. Later, the Coronavirus Study Group of the International Committee on Taxonomy of Viruses renamed the virus “Middle East respiratory syndrome coronavirus” (MERS-CoV) in May 2013 [3].

MERS-CoV is a zoonotic virus that is transmitted from animals that are a reservoir of the virus to humans. Although the source of MERS-COV is not elucidated yet, camels are the most likely source of infection in human [3, 4]. A coronavirus similar to large extent to the one detected in humans has been isolated from camels in Egypt, Oman, Qatar, and Saudi Arabia. Other animals including goats, cows, sheep, water buffalo, swine, and wild birds, have been tested for MERS-CoV and only dromedary camels have evidence of sero-positivity to MERS-COV, supporting the premise that dromedary camels are a likely source of infection in humans [3].

Direct contact with the saliva of infected camels, or consumption of contaminated milk or meat was the suspected transmission route for human MERS-COV infection [4]. Previous study found that the seroprevalence of MERS-CoV was several times higher in persons with regular exposure to camels than in the general population [5, 6]. However, there are some cases infection in which the patients do not have any contact with sick animals or their products. This might be human to human route of transmission.

Only limited numbers of zoonotic diseases have been reported to be transmitted from human to human [7]. The incapability of MERS-CoV to infect animal models like hamsters, mice, and ferrets, indicates the presence of a species barrier. However, an experimental study showed that human cell lines were susceptible to MERS-CoV infection [8], and the reports of human-to-human transmission have increased [9–11]. The modes of human-to-human MERS-CoV spread are incompletely defined [12].

Emergence of the Middle East respiratory syndrome coronavirus (MERS-CoV) has caused significant concern. A total of 635 laboratory-confirmed cases of MERS-CoV infection have been reported globally, including 193 deaths. Cases of MERS-CoV in Saudi Arabia were reported for the first time in September 2012, following the death of a patient due to a severe respiratory illness [13–15]. Little is known regarding the extent of human infection or the degree of detection bias towards more severe cases. If the severe cases currently being detected represent only a small sentinel minority of a much larger population of milder cases (as occurred in the early stage of the 2009 H1N1 pandemic in Mexico) [16], the case-fatality ratio might be substantially lower than what current surveillance data suggest. Conversely, for the severe acute respiratory syndrome (SARS) epidemic of 2003, there was little evidence of the existence of undetected mild or subclinical infections [17–20].

Risk factors for the disease in humans are incompletely understood [21], although MERS is proposed to be a zoonotic disease. Dromedary camels have been implicated due to reports that some confirmed cases were exposed to camels. In the Middle East, confirmed cases of MERS-CoV have arisen as sporadic, familial, or hospital clusters [13, 22–26]. Although human-to-human transmission of MERS-CoV has been identified in many European, African, and Middle Eastern countries, [27–30] a genomic analysis of the Riyadh MERS-CoV isolates suggested that there were three genetically distinct lineages of MERS-CoV; therefore, it was unlikely that the Riyadh infections were the result of a single, continuous chain of human-to-human transmission [15, 31, 32]. A recent study provided further evidence of non-sustainable transmission among humans and suggested that transmission within Saudi Arabia was dependent on contact with an animal reservoir or animal products [14]; however, no animal reservoir has yet been identified. While contact with camels has been reported, these reports were limited to only the primary cases [22, 33–37]. The MERS-CoV was related with a strain of severe acute respiratory syndrome coronavirus (SARS-CoV) that caused an outbreak in 29 countries in 2002–2003. This outbreak was characterized by 8273 cases and 775 deaths, with the majority of cases in Hong Kong [16]. As was determined for the SARS-CoV during its pre-pandemic stage, the MERS-CoV has likely been repeatedly transmitted from an unknown animal host to humans in the past year [17–20].

To obtain effective control of the MERS-CoV outbreak, the MOH of the Kingdom of Saudi summoned a Rapid Response Team (RRT). The RRT was composed of 15 infectious disease doctors and two infection control professionals affiliated with the Korean Society for Infectious Diseases and the Korean Society for Healthcare-associated Infection Control and Prevention. The RRT established national infection control and prevention guidelines for the diagnosis and management of MERS-CoV infections [32, 38–41]. The current study aimed to investigate the demographic characteristics, history of contact with camels or positive cases, mortality, clinical manifestation and comorbidities for confirmed cases of MERS-CoV.

## Methods

### Data collection

All laboratory confirmed MERS-CoV cases who were reported by the Saudi Ministry of Health to WHO from April 23, 2014 to August 31, 2015, were identified. Patient’s charts were reviewed for demographic information, mortality, comorbidities, clinical presentations and health care facility.

### Case definition

A confirmed case was defined as any person with laboratory confirmation of MERS-CoV infection based on positive real-time polymerase chain reaction (PCR) of MERS-CoV in swab samples collected by the MOH in addition to any one of the following clinical definitions: fever (>38 °C), a cough, shortness of breath (SOB), sore throat, vomiting, diarrhea, hemoptysis, chest pain and/or infection, respiratory failure, loss of consciousness, runny nose and any asymptomatic outpatients with a history of contact with positive symptomatic cases and tested positive. Patients provided their signed consent to publication where legal guardian provided consent for a minor.

### Molecular testing

All PCR testing was carried out at the Saudi Ministry of Health MERS-CoV regional laboratory in Riyadh. Respiratory samples were obtained from all patients and submitted to the regional lab to be tested for MERS-COV infection using primers that amplify both the upstream E protein (upE) and ORF1a genes. Samples that tested positive for both upE and Orf 1a gene targets were considered confirmed cases. Each patient was tested at least twice, each on a different day.

### Statistical analysis

Differences were assessed for significance using chi-square goodness of fit and non-parametric binomial tests, where appropriate. Statistical analyses were performed using SPSS, version 21 software (IBM Corp., Armonk, NY, USA). *P* < 0.05 was considered statistically significant.

## Results

**Fig. 1:**
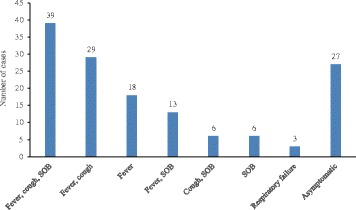
Clinical manifestations of patients with confirmed Middle East respiratory syndrome coronavirus (*n* = 190) between April 23, 2014 and August 31, 2015

As is shown in Table 1, the confirmed male-to-female case ratio was 1.6:1. There were significantly more infections among Saudi patients than among non-Saudi patients (62% versus 37.4%; *P* = 0 · 001; Table 1). Of the 190 patients, 141 (74.2%) had no history of contact with camels or positive cases. A total of 147 (73.2%) patients were aged 20–60 years. The lowest percentage of patients was younger than 20 years of age. Of the 190 patients with confirmed MERS-CoV infection, 69 patients (36.3%) died and 121 patients (63.7%) lived (Table 2). The most common symptoms at presentation were (fever, cough, SOB) (*n* = 39; 20.5%), (fever, cough) (*n* = 29; 15.3%), fever (*n* = 18; 9.5%), (fever, SOB) (*n* = 13; 6.8%), (SOB, cough) (*n* = 6; 3.2%), and Respiratory Failure (*n* = 3; 1.6%) (Fig. 1). Other symptoms were reported less frequently by 5% of patients including chest infection; cold, cough, SOB; cough, diarrhea; cough, SOB, hemaptisis; fever, cough, SOB, vomiting and cough, SOB. All 27 asymptomatic patients (14.2% of 190) with a history of contact with positive symptomatic cases were tested positive (Fig. 1).

**Table 1:** Demographic characteristics of Middle East respiratory syndrome coronavirus cases in Saudi Arabia between April 23, 2014 and August 31, 2015

Variable	Confirmed Cases (*n* = 190)	*P*-value
Sex		0.003
M	116 (61.1)	
F	74 (38.9)	
groups Age		<0.0001
0–20	8 (4.2)	
21–40	71 (37.4)	
41–60	68 (35.8)	
≥ 61	43 (22.6)	
Nationality		0.001
Saudi	119 (62.6)	
Non-Saudi	71 (37.4)	
History of contact		<0.0001
Camels or	1 (0.5)	
positive case	48 (25.3)	
None	141 (74.2)	

Data are n (%)

**Table 2:** Mortality rates of confirmed versus suspected cases of Middle East respiratory syndrome coronavirus between April 23, 2014 and August 31, 2015

Outcome	Confirmed	*P*-value
Survival	121 (63.7)	<0.0001
Death	69 (36.3)	
Total	190	

Data are n (%)

**Fig. 2:**
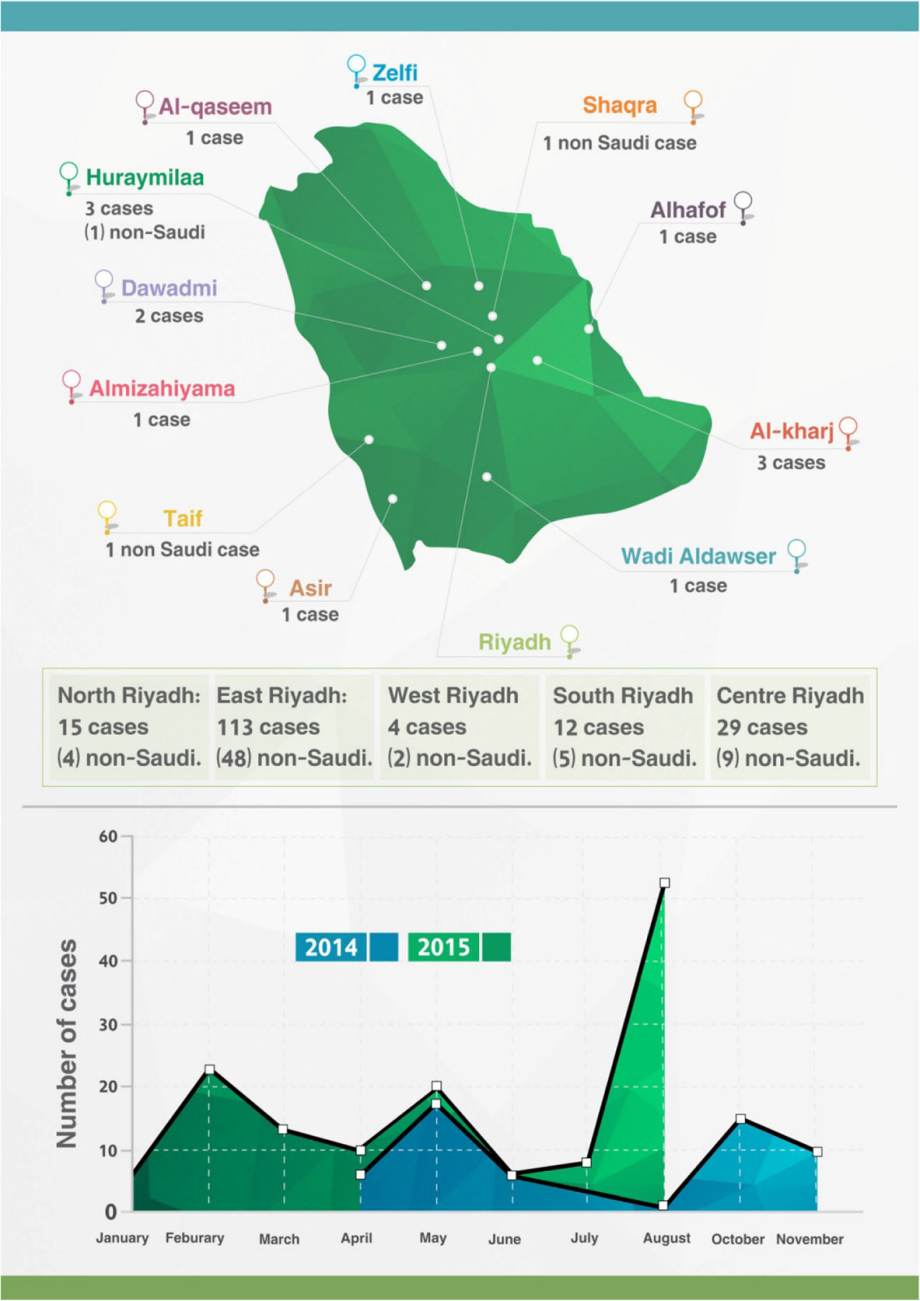
Distributions of Middle East respiratory syndrome coronavirus cases in Saudi Arabia between April 23, 2014 and August 31, 2015

Figure 2 presents frequency and distribution of the confirmed cases that were admitted to health facilities from different provinces in Saudi Arabia. In Riyadh, the majority of cases were reported from the Eastern region of Riyadh (113 cases, (59.5%)). 29 cases (15.3%) from Central region of Riyadh, 15 cases (7.9%) from North region in Riyadh, 12 cases (6.3%) from South region of Riyadh and 4 cases (2.1%) from West region in Riyadh. On the other hand, 16 cases (8.4%) collectively reported from other provinces in Saudi Arabia. Of the 190 patients, 108 (56.8%) had underlying medical disorders, and 82 (43.2%) were previously healthy (Table 3). Diabetes 60 (31.6%), hypertension 54 (28.4%) and bronchial asthma 18 (9.5%) were the most frequent comorbid disorders (Table 3).

**Table 3:** Frequency of comorbidities in patients with confirmed Middle East respiratory syndrome coronavirus

Comorbidities	Patients
Diabetes mellitus	60 (31.6)
Hypertension	54 (28.4)
B. asthma	18 (9.5)
IHD	11 (5.8)
CHF	5 (2.6)
End-stage renal disease	5 (2.6)
Pneumonia	4 (2.1)
Hypothyroidism	4 (2.1)
CVA	3 (1.6)
Liver cirrhosis	2 (1.1)
HF	1 (0.5)
BPH	1 (0.5)
Hodgkin’s lymphoma	1 (0.5)
Chronic obstructive pulmonary disease	1 (0.5)
Sickle cell anemia	1 (0.5)
Liver carcinoma	1 (0.5)
Mitral + aortic valve replacement	1 (0.5)
Acute respiratory distress syndrome	1 (0.5)
Chronic kidney disease	1 (0.5)
Tuberculosis	1 (0.5)
RF	1 (0.5)
CP	1 (0.5)
COPC	1 (0.5)
Lung cancer	1 (0.5)
Anemia	1 (0.5)
Gout	1 (0.5)
Myeloma	1 (0.5)
None	82 (43.2)

Data are n (%)

## Discussion

Of the 190 (116 men and 74 women) confirmed cases of MERS-CoV infection in our study, only a single patient had a history of direct contact with camels and 48 had direct contact with positive cases of MERS-CoV; 141 patients did not have any contact with camels and other sick patients. An analysis of the demographic characteristics of the confirmed cases of MERS-CoV during the 18-month study period demonstrated that only eight cases were patients aged <20 years; 73.2% of the cases were patients aged 21–60 years, and 22.6% of the cases were patients aged >61 years. Notably, a high number (37.4%) of the confirmed cases of MERS-CoV were not of Saudi nationality.

The respiratory clinical manifestations of MERS-CoV infection for all of the patients in the present study were similar to those observed in previous studies of other Saudi patients from different regions in Saudi Arabia. Fever with cough and SOB, then fever with cough were the most common symptoms among patients (20.5% and 15.3%, respectively). These findings are consistent with previous reports [21, 22, 38, 42–47].

Notably, the clinical features of MERS-CoV infection ranged from asymptomatic or mild disease to acute respiratory distress syndrome and multi-organ failure, which is consistent with the findings of previous studies from various countries [33, 34, 40, 43, 47, 48]. To date, only three studies have reported the clinical characteristics and outcomes of patients with MERS-CoV infection who were admitted to the intensive care unit (ICU), which collectively included 34 patients [44–47, 49, 50]. Arabi et al. analyzed data for 12 patients admitted to two ICUs in Riyadh and one in Al-Hasa in the central and eastern parts of the country, respectively [47]. The remaining two reports were both from Jeddah in Western Saudi Arabia. The study by Al-Hameed et al. included eight patients [44], whereas Khalid et al. described the clinical course and outcomes of 14 patients with severe MERS-CoV infection [50].

There is a need to improve the collaboration between scientific researchers, clinical units, and public health communities in order to provide an effective community health service and to prepare for future outbreaks of MERS-CoV, to ultimately decrease the number of new cases [22, 29, 35, 47, 48]. The MERS-CoV outbreak in Saudi Arabia appeared to be reaching controlled levels, with a significant decrease in the number of new cases. Furthermore, there were no new super spreading events that could result in a third epidemic peak. Vigilant monitoring will be crucial to end the MERS-CoV outbreak. The RRT hopes to share their knowledge of the MERS-CoV outbreak with other countries and to cooperate in the prevention of the MERS-CoV outbreak from becoming a global pandemic.

## Conclusion

MERS-CoV is considered an epidemic in Saudi Arabia. The results of the present study showed that the frequency of cases is higher among men than women, in Saudi patients than non-Saudi, and those between 21 to 60 years are most affected. Furthermore, Eastern Region of Riyadh had the highest number of cases. However, further studies are required to determine association of demographic characteristics with mortality, source, and route of infection transmission in Saudi Arabia. We recommend that much more studied are remains to be discovered about MERS-CoV. Improved surveillance, epidemiological research for the development of new therapies and vaccine are important for both human and camels. Further studies are required to gains in terms of better understanding of disease patterns should be maintained to enable the global community to answer the remaining questions about this disease.

## Abbreviations

ICU: Intensive care unit
MERS: the Middle East respiratory syndrome
MOH: Ministry of Health
RRT: Rapid Response Team
SARS: Severe acute respiratory syndrome
WHO: World Health Organization
